# Chloroplast genome of *Calamus tetradactylus* revealed rattan phylogeny

**DOI:** 10.1186/s12863-024-01222-0

**Published:** 2024-03-25

**Authors:** Haibo Zhang, Peng Liu, Yi Zhang, Huayu Sun, Yue Wang, Zhimin Gao, Xin Liu

**Affiliations:** 1 BGI Research, Beijing, 102601 China; 2 School of nursing, Chongqing Medical and Pharmaceutical College, P. R, Chongqing, China; 3 Key Laboratory of National Forestry and Grassland Administration/Beijing for Bamboo and Rattan Science and Technology, Beijing, 100102 China; 4 Institute of Gene Science and Industrialization for Bamboo and Rattan Resources, International Centre for Bamboo and Rattan, Beijing, 100102 China

**Keywords:** *Calamus tetradactylus*, Chloroplast genome, Phylogeny, Comparative genomics

## Abstract

**Background:**

*Calamus tetradactylus*, a species primarily distributed in Vietnam, Laos, and southern China, is highly valued for its utilization as a small-diameter rattan material. While its physical and mechanical properties have been extensively studied, the genomic characteristics of *C. tetradactylus* remain largely unexplored.

**Results:**

To gain a better understanding of its chloroplast genomic features and evolutionary relationships, we conducted sequencing and assembly of the chloroplast genome of *C. tetradactylus*. The complete chloroplast genome exhibited the typical highly conserved quartile structure, with specific variable regions identified in the single-copy region (like *psbF-psbE*, *π* = 0.10327, *ndhF-rpl32*, *π* = 0.10195), as well as genes such as *trnT-GGU* (*π* = 0.05764) and *ycf1* (*π* = 0.03345) and others. We propose that these regions and genes hold potential as markers for species identification. Furthermore, phylogenetic analysis revealed that *C. tetradactylus* formed a distinct clade within the phylogenetic tree, alongside other *Calamus* species, and *C. tetradactylus* was most closely related to *C. walkeri*, providing support for the monophyly of the genus.

**Conclusion:**

The analysis of the chloroplast genome conducted in this study provides valuable insights that can contribute to the improvement of rattan breeding programs and facilitate sustainable development in the future.

**Supplementary Information:**

The online version contains supplementary material available at 10.1186/s12863-024-01222-0.

## Introduction

Chloroplasts in plants result from cyanobacteria and eukaryotic cell symbiosis, converting light energy through photosynthesis. Angiosperms have circular chloroplast genomes containing essential genes for growth. Maternally inherited chloroplast genome has a smaller size and conserved features, facilitating the study of phylogenetics and molecular evolution. Rattan is a climbing plants belonging to the Calamoideae of the Arecaceae family, predominantly found in tropical rainforests [1, 2]. The genus *Calamus*, the largest within Arecaceae, includes approximately 400 species prominently distributed across the Asia-Pacific region [3]. Rattan cane, a non-timber forest produce, is widely utilized in the production of a variety of craft products and furniture [4]. While there has been extensive research on the physical and mechanical properties of rattan species in Southeast Asia since the 1860s [5–7], studies in the field of molecular genetics have been limited. Therefore, genomic information is crucial for improving phylogenetic inference for revealing evolutionary history and genetic relationships. In this study, we applied sequencing technologies to study rattan species of chloroplast genome.

*Calamus tetradactylus*, a slender rattan species, is mainly found in areas north of 23°30′ N, including Guangdong, Guangxi, Fujian, and Hainan, within the *Calamus* genus of the Arecaceae family [8]. *C. tetradactylus* grows over 30 m in height, and it is known for its exceptional quality mechanical strength, making it as one of the most significant commercial rattans [9]. Previous research on *C. tetradactylus* has primarily focused on optimizing the conditions for cultivation but with limited importance given to molecular characterization. Yao et al. [8] analyzed the phylogenetic relationships of around 180 Arecaceae species using chloroplast genomes. However, it did not reveal any differences between the chloroplast genomes of *C. tetradactylus* and other closely related species in addition to lack of its evolutionary position within the plant kingdom. This study aims to elucidate the characteristics of the chloroplast genome in *C. tetradactylus* and highlight its distinctions from other species to provide insights into its taxonomic status within the plant kingdom based on the chloroplast genome.

## Results

### Features of the chloroplast genomes in *C. tetradactylus*

**Fig. 1:**
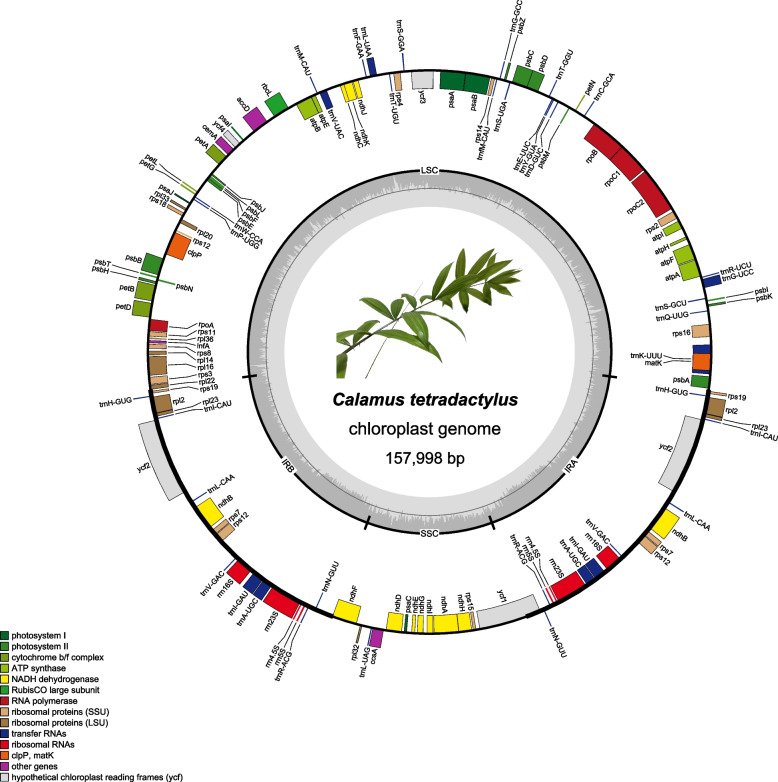
Circular map of chloroplast genome of *Calamus tetradactylus* with annotated genes. The different functional genes groups are shown in different colors, which are shown on the bottom left. The genes transcribed in clockwise and counterclockwise are shown inside and outside of the external circle, respectively. The inner circle represents that the quadripartite structure contains two copies of the inverted repeat (IR) region (IRA and IRB), which separate large single copy (LSC) and small single copy (SSC) region. The dark gray color of inner circle shows the GC content, and AT content in light gray

Complete genome of chloroplast genome of *C. tetradactylus* was obtained through sequencing and assembly. It has a length of 157,998 bp with a typical quadripartite structure (Fig. 1). The genome consists of an 85,760 bp Large Single-Copy (LSC) region, a 17,602 bp Short Single-Copy (SSC) region, and two Inverted Repeat (IR) regions (IRa and IRb) spanning 27,318 bp length. The overall GC content is 37.24%. The LSC, SSC and IR regions has a GC content of 35.25, 31.23, and 42.30% respectively. The *C. tetradactylus* chloroplast genome encodes a total of 132 genes, including 86 protein-coding genes (CDS), 38 transfer RNA genes (tRNA), and eight ribosomal RNA genes (rRNA). The LSC region contains 60 CDS and 21 tRNA genes, while the SSC region contains 12 CDS and a unique tRNA gene (*trnL-UAG*) (Table 1). Duplicated genes in the IR regions include *ndhB*, *rpl2*, *rpl23*, *rps7*, *rps12*, *rps19*, and *ycf2*, and all four rRNA genes and eight of the 38 tRNA genes are duplicated in the IR regions (Table 2). Among the 132 genes, 21 contain introns, with 15 genes having one intron (*atpF*, *rps16*, *rpoC1*, *rpl2*, *rpl16*, *petB*, *petD*, *ndhA*, *ndhB*, *trnA-UGC*, *trnG-UCC*, *trnI-GAU*, *trnK-UUU*, *trnL-UAA*, *trnV-UAC*), and two genes containing two introns (*ycf3* and *clpP*) (Supplemental Table 1).

**Table 1:** Chloroplast genome composition of *C. tetradactylus*

Region	Size (bp)	GC (%)	Genes	CDS	tRNA	rRNA
Genome	157,998	37.24	132	86	38	8
LSC	85,760	35.25	81	60	21	0
SSC	17,602	31.23	13	12	1	0
IRa	27,318	42.30	19	7	8	4
IRb	27,318	42.30	19	7	8	4

**Table 2:** Genes in the chloroplast genome of *C. tetradactylus*

Category	Gene group	Region	Gene name in each region
Genes for photosynthesis	Subunits of photosystem I	LSC	*psaA, psaB, psaI, psaJ*
		SSC	*psaC*
	Subunits of photosystem II	LSC	*psbA, psbB, psbC, psbD, psbE, psbF, psbH, psbI, psbJ, psbK, psbL, psbM, psbN, psbT, psbZ*
	Subunits of ATP synthase	LSC	*atpA, atpB, atpE, atpF, atpH, atpI*
	Subunits of NADH-dehydrogenase	LSC	*ndhC, ndhJ, ndhK*
		SSC	*ndhA, ndhD, ndhE, ndhF, ndhG, ndhH, ndhI*
		IRa & IRb	*ndhB*
	Subunits of cytochrome b/f complex	LSC	*petA, petB, petD, petG, petL, petN*
	Subunits of rubisco	LSC	*rbcL*
Self replication	Large subunit of ribosome	LSC	*rpl14, rpl16, rpl20, rpl22, rpl33, rpl36*
		SSC	*rpl32*
		IRa & IRb	*rpl2, rpl23*
	Small subunit of ribosome	LSC	*rps2, rps3, rps4, rps8, rps11, rps14, rps16, rps18*
		SSC	*rps15*
		IRa & IRb	*rps7, rps12, rps19*
	DNA dependent RNA polymerase	LSC	*rpoA, rpoB, rpoC1, rpoC2*
	Ribosomal RNAs	IRa & IRb	*rrn4.5S, rrn5S, rrn16S, rrn23S*
	Transfer RNAs	LSC	*trnP-UGG, trnW-CCA, trnM-CAU, trnV-UAC, trnF-GAA, trnL-UAA, trnT-UGU, trnS-GGA, trnfM-CAU, trnG-GCC, trnS-UGA, trnT-GGU, trnE-UUC, trnY-GUA, trnD-GUC, trnC-GCA, trnR-UCU, trnG-UCC, trnS-GCU, trnQ-UUG, trnK-UUU*
		SSC	*trnL-UAG*
		IRa & IRb	*trnA-UGC, trnH-GUG, trnI-CAU, trnL-CAA, trnV-GAC, trnI-GAU, trnR-ACG, trnN-GUU*
Other genes	Maturase	LSC	*matK*
	Protease	LSC	*clpP*
	Envelop membrane protein	LSC	*cemA*
	Subunit of Acetyl-CoA-carboxylase	LSC	*accD*
	c-type cytochrom synthesis gene	SSC	*ccsA*
	Translational initiation factor	LSC	*infA*
Unkown	Conserved open reading frames	LSC	*ycf3, ycf4*
		SSC	*ycf1*
		IRa & IRb	*ycf2*

### Phylogeny revealed through chloroplast genome comparison

**Fig. 2:**
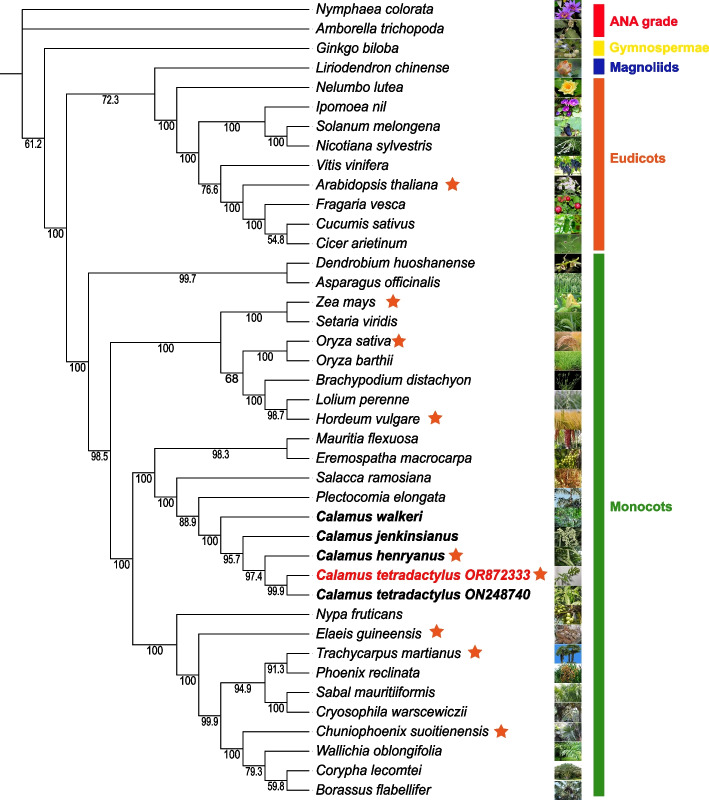
Phylogenetic tree based on the chloroplast gene sequences of 42 plant species. The species with red star marks are selected for the comparison of IR/SC boundary regions in Fig. 3. The genbank accession number are listed in Supplemental Table 3

The chloroplast genome, one of the three genetic systems in green plants, has gained significant attention in evolutionary studies due to its maternal inheritance with relatively lower mutation rate. Chloroplast genome can yield more reliable results for determining phylogenetic relationships among green plants, thus complete chloroplast genomes hold great value in determining the phylogenetic relationships among closely related taxa and enhancing our understanding of genetic evolution of plant species. Thus, we conducted a detailed study that involved the selection of 41 diverse plant species representing major clades of land plants (Fig. 2). This study also aimed to construct a highly informative phylogenetic tree based on the meticulous analysis of chloroplast gene sequences. To achieve this, we incorporated a wide range of taxa, consisting of one representative from Gymnospermae, two from ANA grade, one from Magnoliids, 10 from Eudicots, and 27 species from Monocots. By including such a diverse array of taxa, we aimed to capture the full spectrum of plant diversity to understand the evolutionary relationships within and between different clades. The resulting phylogenetic tree revealed a remarkable pattern of distinct clades, each representing a unique evolutionary lineage. Notably, the prominent Arecaceae family was found to be clustered within the Monocots clade. Delving deeper into the Arecaceae clade, we observed the formation of two separate clades, one of which included Calameae and other tribes. Our analysis supported the hypothesis that *C. tetradactylus* shares a close evolutionary relationship with other monocots. In fact, our phylogenetic tree unequivocally positioned *C. tetradactylus* as a sister species to the rest of the monocots, reinforcing the notion of shared ancestry and providing compelling evidence for its placement within the broader monocot lineage (Fig. 2). These results are generally consistent with the previous study [8].

**Fig. 3:**
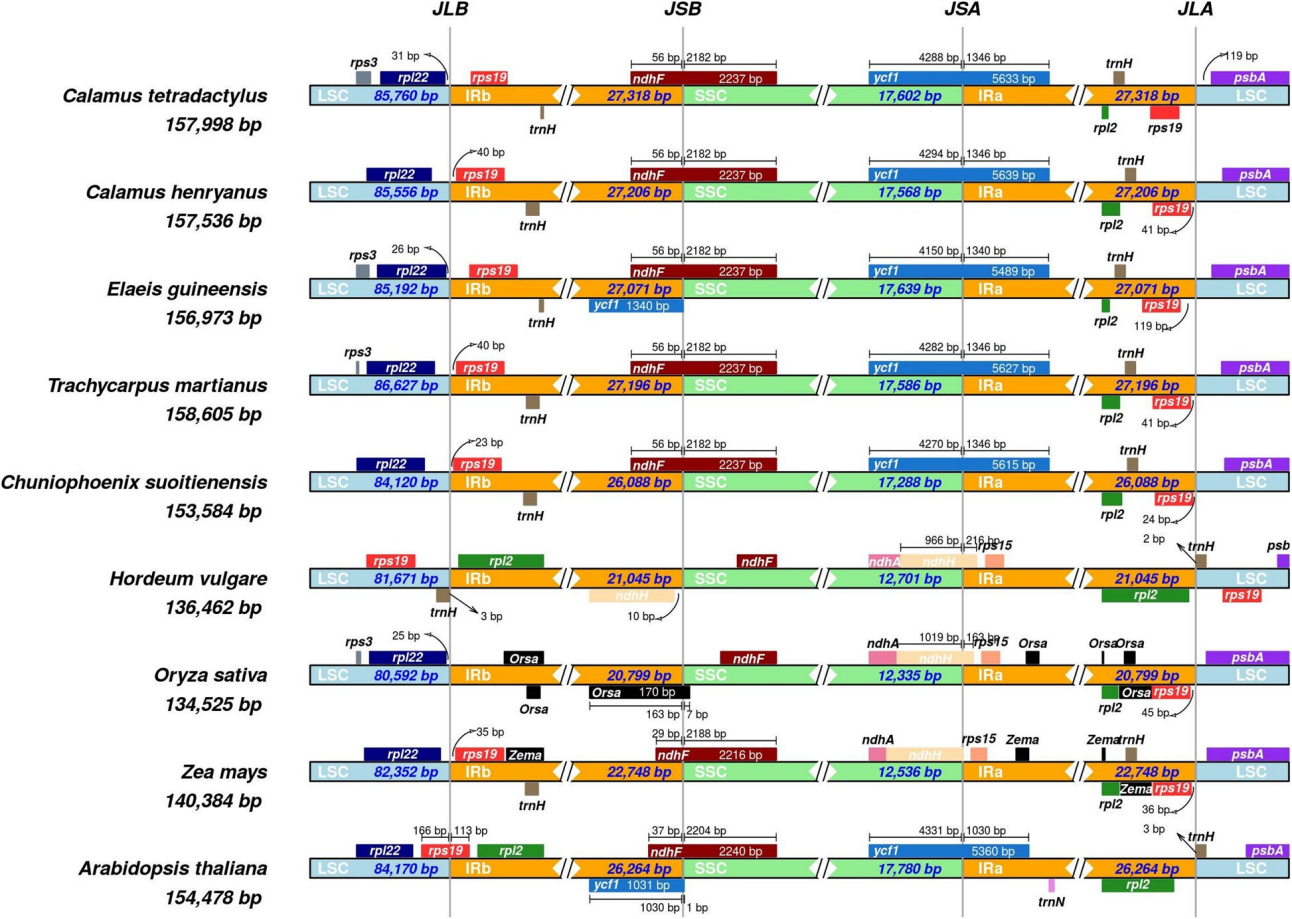
The comparison of IR/SC boundary regions of chloroplast genomes. The thin vertical lines represent the junction of each region, and the map displays information about the genes near the junction. LSC, Large single copy; SSC, Small single copy; IRa and IRb, inverted repeats. JLB, junction between LSC and IRb; JSB, junction between SSC and IRb; JSA, junction between SSC and IRa; JLA, junction between LSC and IRa

In addition, our phylogenetic tree includes four species from the Calaminae subfamily, allowing us to uncover their close relationship and identify *C. tetradactylus* as the species most closely related to *C. walkeri* (Fig. 2). This finding aligns with the results obtained from morphological classification [10]. Notably, we observed differences in the *ycf1* gene within the JSB region between *C. tetradactylus* and *Elaeis guineensis*, reflecting their evolutionary divergence (Fig. 3). Furthermore, variations in the *rps3* gene within the LSC region and the *rps19* gene at the IR-LSC boundary revealed the genetic relationship between *C. tetradactylus*, *Trachycarpus martianus*, and *Chuniophoenix suoitienensis*. These findings lay the foundation for further investigations into the intriguing evolutionary history of plants and provide valuable insights into the genetic diversity and adaptation of *C. tetradactylus* and other related monocot species.

### Comparison of *C. tetradactylus* chloroplast genome

The chloroplast genome of *C. tetradactylus* exhibits a typical quadripartite structure with four borders, including the JLB and JLA, which is the junction between LSC and IRb or IRa, the JSB and JSA, which is the junction between SSC and IRb or IRa. In our study, we compared the conserved regions of the chloroplast genome of *C. tetradactylus* with eight other species, including four species of Arecaceae, three species of Poaceae, and *Arabidopsis thaliana* (Fig. 3). Compared to the other species, the chloroplast genome of *C. tetradactylus* is similar to that of the four Arecaceae species, but larger than that of Poaceae species (*Hordeum vulgare*, *Oryza sativa*, *Zea mays*) and *A. thaliana*. Notably, the LSC region of *C. tetradactylus*, with a length of 85,760 bp, is longer than that of most other plants with a sequence length (80,592 bp ~ 85,556 bp), except for *T. martianus* (86,627 bp). Additionally, the IR regions of *C. tetradactylus* have greater length of 27,318 bp compared to the other eight species. The SSC region of *C. tetradactylus*, with 17,602 bp, is smaller than that of *E. guineensis* (17,639 bp) and *A. thaliana* (17,780 bp), but longer than that of the other six species. The gene content and arrangement in the Arecaceae species are similar across the four regions. The LSC region contains the *rpl22* and *psbA* genes, while the *rps19* and *rpl2* genes are distributed in the IR region. The JSB and JSA regions are spanned by the *ndhF* and *ycf1* genes, respectively, and their lengths reflect variations in these regions. In comparison, the *rps19* gene of *H. vulgare* is in the LSC region, but in *A. thaliana*, it spans the JLB region. The *ndhF* genes of *H. vulgare* and *O. sativa* are much smaller than those of *C. tetradactylus* and are only found in the SSC region. The locations of *ndhA*, *ndhH*, and *rps15* genes in Poaceae species (*H. vulgare*, *O. sativa*, *Z. mays*) correspond to the position of the *ycf1* gene in *C. tetradactylus* (Fig. 3). In summary, *C. tetradactylus* has a unique chloroplast genome with distinct borders and larger size compared to other species. It shares similarities with Arecaceae species but differs in gene locations and lengths.

### Sequence variations in regions and genes

**Fig. 4:**
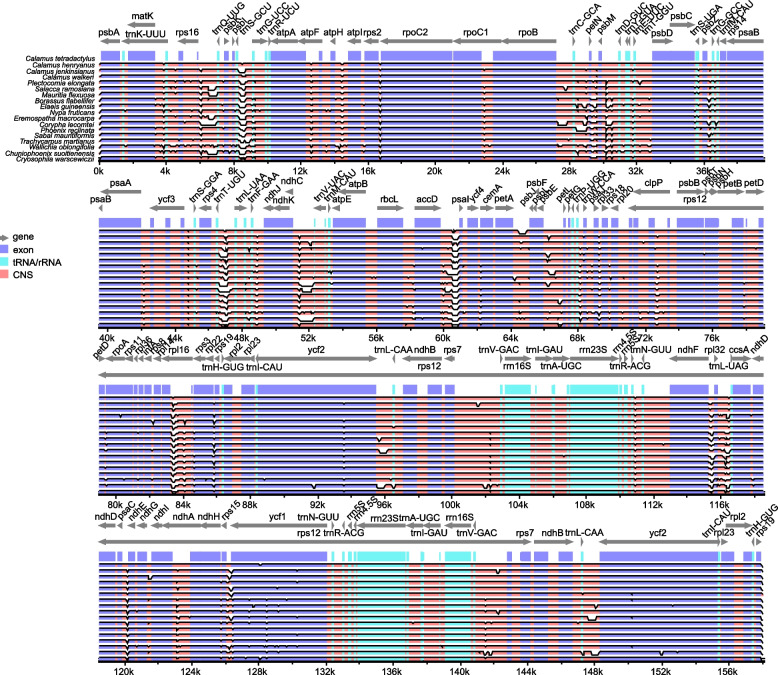
Visualized alignment of the *C. tetradactylus* chloroplast genome sequences with annotations using mVISTA. Each horizontal lane displays the percent of conservation identify with *C. tetradactylus* as reference. The x-axis represents the aligned base sequences, and y-axis represents percent pairwise identity within 50–100%

**Fig. 5:**
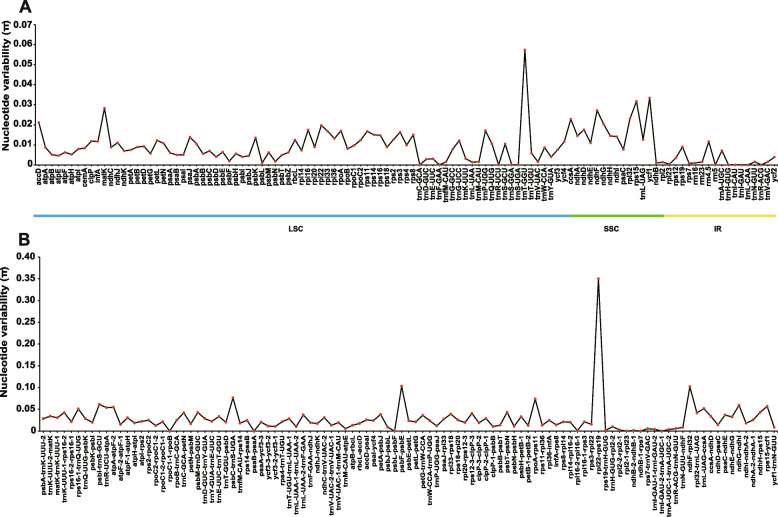
The nucleotide diversity (*π*) values of chloroplast genome. **A** The Pi value of different genes in LSC, SSC and IR regions. **B** The Pi value of non-coding region. The x-axis represents the name of gene (**A**) or non-coding region (**B**). The y-axis represents the value of Pi

Although chloroplast genomes are generally conserved among different species, they generally exhibit sequence variations that may hold a variety of biological significances [11]. These variations are effectively utilized as genetic markers to distinguish between different species [12, 13]. Thus we compared the *C. tetradactylus* chloroplast genome to those of the related species to identify the sequence variations. The chloroplast genomes of 18 Arecaceae species, including species from Calamoideae, Nypoideae, Coryphoideae, and Elaeidinae subfamilies, were compared with the *C. tetradactylus* chloroplast genome as the reference (Fig. 4). While we observed only a few minor variations in the coding sequences (i.e., *accD*, *ycf2* and *ycf1*), a substantial number of divergences were detected in the conserved non-coding sequence (CNS) regions. Interestingly, the IR region exhibited the lowest degree of variation, indicating its high evolutionary conservation. In contrast, the LSC region displayed the highest variation across the chloroplast genome suggesting it to be more dynamic. Among the different gene types, tRNA/rRNA genes were found to be the most conserved, as no significant variations were observed (Fig. 4). To measure the variation of nucleotide sequences among different species, we calculated the nucleotide diversity (*π*) values (Fig. 5). Highly variable regions can serve as potential DNA markers for population genetics studies. In a global comparison of homologous genes from different species, we found that the nucleotide diversity in the LSC and SSC regions was higher compared to the IR regions. Specifically, the *trnT-GGU* gene in the LSC region exhibited the highest diversity, with a maximum *π* value of 0.0575 (Fig. 5A). The *trnT-*GGU gene with high diversity was also found in Geraniaceae, which may be related to pseudogenization associated with an insertion event in the 5′ acceptor stem [14]. In the SSC region, the *ycf1* gene displayed the largest diversity, with a *π* value of 0.0335 (Fig. 5A). The section of *ycf1* in the SSC region has been predicted to have high nucleotide diversity and has been used in molecular systematics at the species level in angiosperm [15, 16].

Furthermore, through alignment of the non-coding regions, we identified 12 highly variant regions (*π* > 0.05) that were identified as the main divergent regions (Fig. 5B). These regions include *rpl22-rps19*, *psbF-psbE*, *ndhF-rpl32*, *psbC-trnS-UGA*, *rpoA-rps11*, *psbI-trnS-GCU*, *ndhG-ndhI*, *rps15-ycf1*, *atpA-atpF-2*, *trnR-UCU-atpA*, *trnL-UAG-ccsA*, and *rps16–1-trnQ-UUG*. Detailed information can be found in Supplemental Table 2. Highly variable loci as SNPs in the chloroplast genome can be used as DNA barcodes for identifying plants. The comparison of the whole chloroplast genome among *Bambusa* species has found that the *rpl16* gene and *psbA-trnH* region could be used to identify *Bambusa subgenera* [17]. DNA derived from the chloroplast genome can be used to identify similar species and is also valuable to enhance the transfer of useful traits [18]. In our study, the previously mentioned nine highly variable genes could be used as potential DNA markers for taxonomic studies of *Calamus*.

The *ndh* genes, which encode subunits of NADH dehydrogenase involved in photosynthesis, play a crucial role in chloroplast function. These ndh proteins assemble into the photosystem I complex, facilitating electron transport within chloroplasts and promoting chlorophyll respiration. In our study, we identified three highly variable ndh genes (*ndhf*, *ndhG*, and *ndhD*) with π values exceeding 0.15 (Supplemental Table 2). It is worth noting that the composition of chloroplast *ndh* genes can differ among autotrophic plants, impacting their function [19–23]. Additionally, we observed significant variability in certain genes within the *rpl* gene family (*rpl32*, *rpl22*, *rpl16*, and *rpl33*) and *rps* gene family (*rps15*, *rps11*, *rps3*, *rps8*, and *rps14*) (Supplemental Table 2). These highly variable sequences can serve as DNA markers for genetic diversity analysis and provide essential DNA barcoding information for species identification. Overall, our findings revealed high levels of genetic diversity and evolutionary dynamics of Arecaceae species, particularly among *ndh* gene family, as well as certain genes within the *rpl* and *rps* gene families.

## Discussion

The chloroplast genome structure, length, and gene content are typical and highly conserved among most terrestrial plants. In our study, we successfully assembled the complete chloroplast genome of *C. tetradactylus*, which spans 157.998 kb and closely resembles that of its closely related species, *C. walkeri* (Fig. 2). However, notable differences were observed between the chloroplast genome of *C. tetradactylus* and other selected species (Figs. 1 and 3). A previous study on five Epimedium species also reported variations in chloroplast genome length among species, attributing these differences could be due to contraction and expansion of genes at the boundaries of the inverted repeat (IR) and small single-copy (SSC) regions [24]. Our findings also indicate that the primary reason for variation in length in the chloroplast genome is the contraction and extension of the IR-LSC and IR-SSC boundaries as reported in many angiosperms [25, 26]. Interestingly, despite *C. tetradactylus* and other Poaceae species (*H. vulgare*, *O. sativa*, and *Z. mays*) belonging to the Monocots group, significant differences in gene length, structure, and genotype were observed in both the junction of the small single-copy region and the junction of the inverted repeat region. The junction of the small single-copy region (JSA) exhibited greater variability, indicating the highest variation in genotype across the chloroplast genomes between *C. tetradactylus* and Poaceae species. The alterations in *ndhF* and *ycf1* sequences may be attributed to the expansion and contraction of the junction of the small single-copy region and the junction of the inverted repeat region in plants, respectively (Fig. 3) [27, 28]. Using the complete chloroplast genome of *C. tetradactylus* that we assembled, we conducted an analysis to determine the phylogenetic relationships among closely related species of *C. tetradactylus* (Figs. 4 and 5). In particularly, we identified specific regions and genes, such as *ycf1*, *rps3*, and *rps19*, that are associated with species divergence. We propose that these regions and genes be further utilized for more detailed phylogenetic analysis, among closely related species and also within populations of a single species. While the current chloroplast genome provides valuable genetic resources for understanding the ecologically and economically important *C. tetradactylus* species, future studies focusing on establishing the complete nuclear genome would greatly enhance our understanding, applications, and advancements related to genetics and molecular breeding of *C. tetradactylus*.

## Conclusions

In this study, we report the complete sequence, assembly, and annotation of the chloroplast genome of *C. tetradactylus*. Our study also reveals the complete chloroplast structure, sequence length variations of the inverted repeat (IR) boundary, single nucleotide polymorphism in addition to elucidation of phylogenetic relationships across the plant kingdom using representative species. Through genome annotation analysis, we confirmed that the chloroplast genome of *C. tetradactylus* follows the typical quadripartite structure as reported in other species. Additionally, we identified several variable regions that hold possible applications as molecular markers. The constructed phylogenetic tree, utilizing 41 chloroplast genomes, provided clear insights into the genetic and evolutionary relationships. Our findings are expected to contribute to future endeavors such as species identification, construction of evolutionary relationships, breeding programs, and sustainable development initiatives in genetic improvement of *C. tetradactylus*.

## Materials and methods

### Experimental materials and sequencing

*C. tetradactylus* plant is grown in the plantation of International Center for Bamboo and Rattan, located in Beijing. Fresh leaves, without signs of pests and disease, were collected and snap-frozen in liquid nitrogen, then stored at − 80 °C until DNA extraction. Total DNA was extracted by the modified CTAB method [29]. DNA quality was measured using a Nanodrop spectrophotometer, and DNA integrity was detected by agarose gel electrophoresis. This study utilized Single-Tube Long Fragment Reads (stLFR) technology to sequence the genome of *C. tetradactylus* [30]. The libraries of stLFR were constructed following the protocol of the MGIEasy stLFR Library Prep Kit (MGI, Shenzhen, China), and then sequenced on MGISEQ-2000 (MGI, Shenzhen, China) at the Beijing Genomics Institution (BGI, Shenzhen, China).

### Chloroplast genome assembly and annotation

In order to acquire clean data, the raw data were then trimmed and filtered using SOAPnuke v2.0 with the parameters -q 33 -y -p -M 2 -f − 1 -Q 10 [31]. Then, the chloroplast DNA was assembled to a circular genome using the organelle genome assembly program GetOrganelle v1.7.7 with the parameters -R 15 -k 21, 45, 65, 85 -F embplant pt. [32]. Geneious v8.0.4 was used to manually edit the assembled genomes for sequence improvement [33], after the genomes were automatically annotated using the online program CPGAVAS2, the previous *C. tetradactylus* chloroplast genome (ON248740) was used as a reference sequence (http://47.96.249.172:16019/analyzer/annotate) [34]. The online program OGDRAW v1.3.1 (https://chlorobox.mpimp-golm.mpg.de/OGDraw.html) was used to create the chloroplast genome maps [35], and tRNAscan-SE 2.0 (http://lowelab.ucsc.edu/tRNAscan-SE/) was used to confirm the correctness of tRNA annotations with default search mode [36].

### Phylogenetic analysis

We acquired 41 chloroplast genomes from NCBI in order to better comprehend the evolutionary structure of the *C. tetradactylus* (see Supplemental Table 3 for a detailed information list). This study included a total of 41 species, comprising 33 additional species and 8 species from the Calaminae family. The HomBlocks workflow was used to align the chloroplast genome sequences, and Maximum Likelihood (ML) were used for the phylogenomic study [37]. ModelFinder determined that GTR + F + I + I + R3 was the best-fit nucleotide substitution model [38]. IQ-TREE v2.0.5 was used to reconstruct the ML tree [39]. With 1000 ultrafast bootstrap repetitions, the ML tree’s branch support was evaluated. The online tools iTOL (https://itol.embl.de/) was used to visualize the phylogenetic relationships [40].

### Sequence alignment analysis

To ascertain the genomic structure, gene content, genome size, and repeat variations, we compared eight species of Calaminae and 10 other species, including both monocots and dicots. First, the chloroplast genome sequences were aligned using the shuffle-LAGAN mode in mVISTA (https://genome.lbl.gov/vista/mvista/submit.shtml) [41], with *C. tetradactylus* as the reference. Subsequently, the LSC, SSC, and IR boundaries genes of the chloroplast genomes of two Calaminae species and six common monocot and dicot plant species were analyzed and visualized using IRscope software (https://irscope.shinyapps.io/irapp/) [42].

### Sequence polymorphism analysis

In order to explore the extent of sequence variation in genes and intergenic regions, we compared sequence polymorphism of 18 species in Fig. 4. The sequences of genes and intergenic regions, and all homologous genes were extracted using Python scripts. Then the sequences of homologous genes from different species were aligned globally using Mafft (v7.505) by automatic mode [43]. Finally, the software DnaSP6 [44] was used to compare the aligned sequences for the calculation of nucleic acid diversity and to obtain the value of π.

### Supplementary Information

**Supplementary material 1.**

## Data Availability

The datasets analyzed in this article are available in the GenBank of NCBI, and the complete chloroplast genome sequence of C. tetradactylus is deposited in CNGB Sequence Archive (CNSA) of China National GenBank DataBase (CNGBdb) with accession number CNA0072950. The other accession numbers for the remaining datasets analyzed in this study are listed in the Supplemental Table 3.

## Abbreviations

kbp: Kilo-base pairs
IR: Inverted repeat regions
LSC: Large single-copy region
SSC: Small single-copy region
rRNAs: Ribosomal RNAs
tRNAs: Transfer RNAs
stLFR: Single-Tube Long Fragment Reads
PE: Paired-end
ML: Maximum likelihood
CDS: Protein-coding genes
JLB: Junction between LSC and IRb
JSB: Junction between SSC and IRb
JSA: Junction between SSC and IRa
JLA: Junction between LSC and IRa
CNS: Conserved non-coding sequence
